# ALS-causing mutations in profilin-1 alter its conformational dynamics: A computational approach to explain propensity for aggregation

**DOI:** 10.1038/s41598-018-31199-7

**Published:** 2018-08-30

**Authors:** Mahmoud Kiaei, Meenakshisundaram Balasubramaniam, Vivek Govind Kumar, Robert J. Shmookler Reis, Mahmoud Moradi, Kottayil I. Varughese

**Affiliations:** 1 Department of Pharmacology and Toxicology, University of Arkansas for Medical Sciences, Little Rock, AR 72205 USA; 2 Department of Geriatrics, University of Arkansas for Medical Sciences, Little Rock, AR 72205 USA; 3 Department of Neurology, University of Arkansas for Medical Sciences, Little Rock, AR 72205 USA; 4 McClellan Veterans Medical Center, Central Arkansas Veterans Healthcare Service, Little Rock, AR 72205 USA; 5 Department of Chemistry and Biochemistry, University of Arkansas at Fayetteville, Fayetteville, AR 72701 USA; 6 Department of Physiology and Biophysics, University of Arkansas for Medical Sciences, Little Rock, AR 72205 USA

## Abstract

Profilin-1 (PFN1) is a 140-amino-acid protein with two distinct binding sites―one for actin and one for poly-L-proline (PLP). The best-described function of PFN1 is to catalyze actin elongation and polymerization. Thus far, eight DNA mutations in the *PFN1* gene encoding the PFN1 protein are associated with human amyotrophic lateral sclerosis (ALS). We and others recently showed that two of these mutations (Gly118Val or G118V and Cys71Gly or C71G) cause ALS in rodents. *In vitro* studies suggested that Met114Thr and Thr109Met cause the protein to behave abnormally and cause neurotoxicity. The mechanism by which a single amino acid change in human PFN1 causes the degeneration of motor neurons is not known. In this study, we investigated the structural perturbations of PFN1 caused by each ALS-associated mutation. We used molecular dynamics simulations to assess how these mutations alter the secondary and tertiary structures of human PFN1. Herein, we present our *in silico* data and analysis on the effect of G118V and T109M mutations on PFN1 and its interactions with actin and PLP. The substitution of valine for glycine reduces the conformational flexibility of the loop region between the α-helix and β-strand and enhances the hydrophobicity of the region. Our *in silico* analysis of T109M indicates that this mutation alters the shape of the PLP-binding site and reduces the flexibility of this site. Simulation studies of PFN1 in its wild type (WT) and mutant forms (both G118V and T109M mutants) revealed differential fluctuation patterns and the formation of salt bridges and hydrogen bonds between critical residues that may shed light on differences between WT and mutant PFN1. In particular, we hypothesize that the flexibility of the actin- and PLP-binding sites in WT PFN1 may allow the protein to adopt slightly different conformations in its free and bound forms. These findings provide new insights into how each of these mutations in PFN1 might increase its propensity for misfolding and aggregation, leading to its dysfunction.

## Introduction

Amyotrophic lateral sclerosis (ALS) is a progressive and fatal degenerative disorder of motor neurons. Studies of mouse models carrying ALS mutations (e.g., *SOD1, TARDP-43*, and *FUS*) have significantly expanded our understanding of ALS over the last 20 years. Multiple cellular pathologies (e.g., aggregation of mutant SOD1, TDP43, FUS, and ubiqulin 2; mitochondrial dysfunction; neuroinflammation; and oxidative damage) lead to the progressive loss of motor neurons, resulting in the paralysis and death of voluntary muscles. Available preclinical and clinical models have not led to any interventions that block motor-neuron death or slow the progression of ALS. Further studies are needed to identify the mechanism by which these mutations cause disease and to expedite the development of effective treatments for people with ALS.

The new mutant PFN1 mouse models for ALS that we have created based on familial ALS (fALS) linked PFN1 is an indispensable tool for studying the gene-specific and ALS disease-specific mechanisms of motor neuron degeneration^1^. Eight mutations in *PFN1* were found to be associated with fALS^1–5^. All of the *PFN1* mutations, except E117G, are specific to a subset of fALS patients^6–8^, as are the SOD1 and TDP-43 mutations. Interestingly, *PFN1* mutations are also found in cases of sporadic ALS (sALS) (e.g., a novel nonsynonymous p.R136W mutation identified in a Chinese female with early-onset sALS)^9^. Mutations in *PFN1* represent a novel mechanism underlying ALS, evidenced by the linkage of common polymorphisms in PFN1, tubulin A4A, dynactin, peripherin, and neurofilament genes to ALS^10–14^. Importantly, the discovery of *PFN1* mutations in ALS^2,3^ has led to the development of PFN1 mouse models of the disease^1,5^. These models prove that mutations in *PFN1* associated with ALS are causal for the disease and provide an extraordinary opportunity to study how *PFN1* mutations confer neurotoxicity.

Here, we examined in detail two of the eight *PFN1* mutations associated with ALS (T109M and G118V), and our study sheds light on how these mutations cause ALS and whether there are two different mechanisms at play. The crystal structure of bovine PFN1 in complex with actin and poly-L-proline (PLP), shows that the G118V mutation is located within the actin-binding site, and the T109M mutation near the PLP-binding site. We postulate that the mutation in the actin-binding motif affects the interaction between profilin-1 and actin and dysregulates the dynamics of actin polymerization. On the other hand, we predict that mutations in the PLP-binding motif affect the interaction between PFN1 and PLP and many other ligands binding at this site^15–17^.

PFN1 is a key enzyme in actin polymerization and its dynamics are critically important for all basic cellular activities. We are interested in understanding the structural changes in PFN1 that are caused by ALS-associated mutations. In neurons, proper PFN1 function is essential during development and is implicated in the formation and maintenance of the neuronal cytoskeleton, growth-cone formation, synaptogenesis, synaptic activities, shape and morphological dynamics, and the growth of dendrites and axons. Additionally, the actin-binding and PLP-binding motifs are critical for the multiple functions of PFN1, including actin elongation, polymerization, and dynamics, as well as for interactions with diverse ligands and binding partners (over 50 of which have been described)^15–17^. Although PFN1 was discovered 30 years ago and has been studied extensively, its potential role in motor neuron disease generated renewed interest in its biology. We hypothesize that the ALS-linked mutations in PFN1 disrupt *de novo* structural dynamics for PFN1 tertiary and quaternary assembly. This would disrupt actin polymerization and other *de novo* functions of PFN1, which may have catastrophic effects on the central nervous system or peripheral neurons and glia.

To explore this hypothesis and to understand the structural, folding, and binding perturbations that may account for PFN1 neurotoxicity, as well as to pinpoint the biophysical changes caused by mutations in the *PFN1* gene, we have analyzed the structure of profilin-1 mutants *in silico*. We have investigated the biophysical properties of the two mutants, G118V and T109M, using *in silico* techniques, as these mutants are implicated in causing ALS based on clinical and pathologic evidence and experiments employing cellular and mouse models of ALS^1,2,5,18–20^. We postulate that these mutations may cause neurotoxicity via two distinct pathways, changes in actin dynamics (G- to F-actin polymerization) and cellular signaling pathways (e.g., PIP2 pathway), via PLP-binding and interactions that require further study. We and others have recently provided evidence *in vivo* and *in vitro* that the G118V mutation disrupts actin binding and reduces the F-actin to G-actin ratio, suggesting that impairment of actin polymerization and of other *de novo* functions of PFN1 is the basis for neuronal death^1,2,5^. Additionally, more recent reports from *in vitro* studies provide evidence that the T109M mutation disrupts PLP binding^20^.

In this study, we have generated and analyzed new data on the alteration and perturbation of the 3-D structure of PFN1 protein caused by G118V and T109M mutations to unravel how a single amino acid substitution affects the tertiary structure and properties of PFN1. The G118V mutation reduces the flexibility and increases the hydrophobicity of the polypeptide chain. We reason that these changes are responsible for the misfolding and aggregation of mutant PFN1 and the basis for ultimately blunting actin polymerization. The T109M mutation appears to distort the PLP-binding site, making it sterically unfavorable for PLP binding. For these studies, we utilized the crystal structure of the ternary complex of PFN1 in complex with actin and PLP (PDB code: 2PAV) www.rcsb.org/structure/2pav ^21^. PFN1 binding to actin and PLP plays an important role in its activity. Profilin–actin complex formation is essential for maintaining sufficient levels of actin monomer for polymerization. Recruitment of the profilin–actin complex for polymerization is accomplished through the binding of the last poly-Pro segment of vasodilator-stimulated phosphoprotein (VASP) to the profilin–actin complex^17,21–23^. Thus, impairment of either of these interactions would disrupt the polymerization and growth of the actin filament.

## Results

### *In silico* study of structural changes due to mutations in PFN1

We simulated and analyzed the structural changes caused by ALS-associated missense mutations in *PFN1* to gain insights into how the altered properties of PFN1 protein might compromise neuronal function and/or survival. A single amino-acid substitution, arising from a missense mutation, can alter the local environment and affect intra-molecular interactions of the residues resulting in conformational changes that are usually local, but that can also be allosteric (affecting remote areas of the molecule). A missense mutation can perturb the molecular structure rather subtly, yet disrupt binding to an associated protein or other ligand. The goal of this study was to simulate and analyze the mutation-induced structural changes and their consequences for diverse interactions.

**Figure 1:**
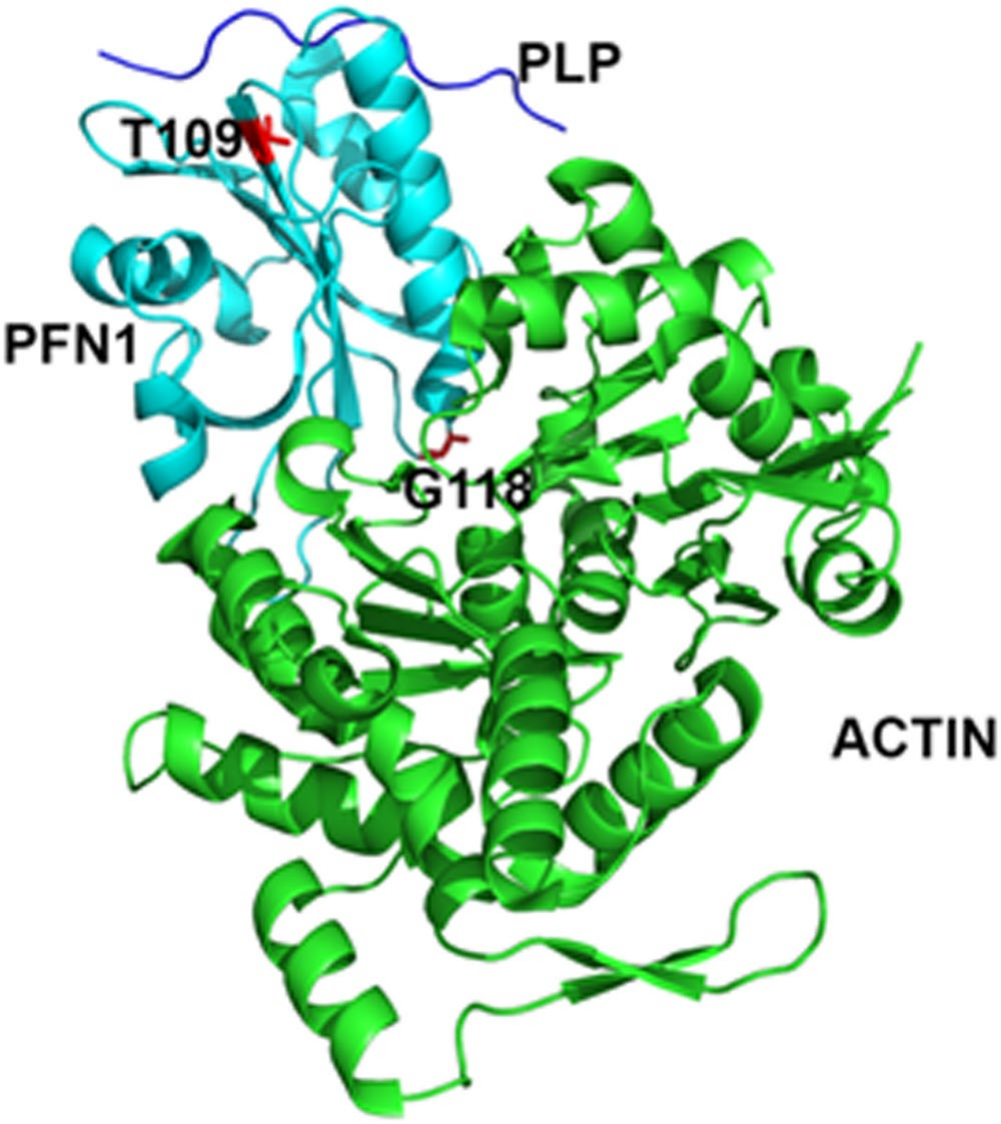
Crystal structure of PFN1 complexes with actin and poly-L-proline peptide (PLP) fragment. The actin-binding site is on the lower part of PFN1 (as shown), while the PLP-binding site is on the top part of PFN1 where PLP is shown in dark blue. PFN1, cyan; PLP, dark blue; and actin, green. The sites of PFN1 mutations T109 and G118 are indicated.

We began with the previously determined crystal structure of PFN1 complexed with an actin fragment and a PLP (PDB code: 2PAV)^21^ (Fig. 1). Using *in silico* modeling techniques^24,25^, we derived structural models of the two mutated PFN1 proteins and predicted the consequences of these mutations (G118V and T109M) on the structure and function of PFN1 in complex with actin or PLP and in its isolated form.

### Glycine 118 to valine mutation

*In vivo* studies and computer modeling suggest that this mutation likely decreases the solubility and aggregation of PFN1^1,19^. Glycine is the smallest nonpolar, hydrophobic amino acid, with only hydrogen as its R group. Gly118 is located in a loop that connects a β-strand to the fifth and last α-helix of the protein (Fig. 1). When PFN1 is co-crystalized with actin, the N-terminus of the helix and adjacent portion of the loop sit in a surface pocket of actin. The crystal structure shows that Gly118 is the first residue of the segment that directly interacts with actin through a hydrogen bond. We conjectured that replacing glycine with valine might disrupt interactions with actin. Both valine and glycine are similarly nonpolar amino acids, but valine carries a larger R group, CH-(CH_3_)_2_. We sought to test this hypothesis while also assessing other possible impacts on structure that might cause misfolding and lead to further anomalies downstream.

### Effect of G118V mutation on conformation

**Figure 2:**
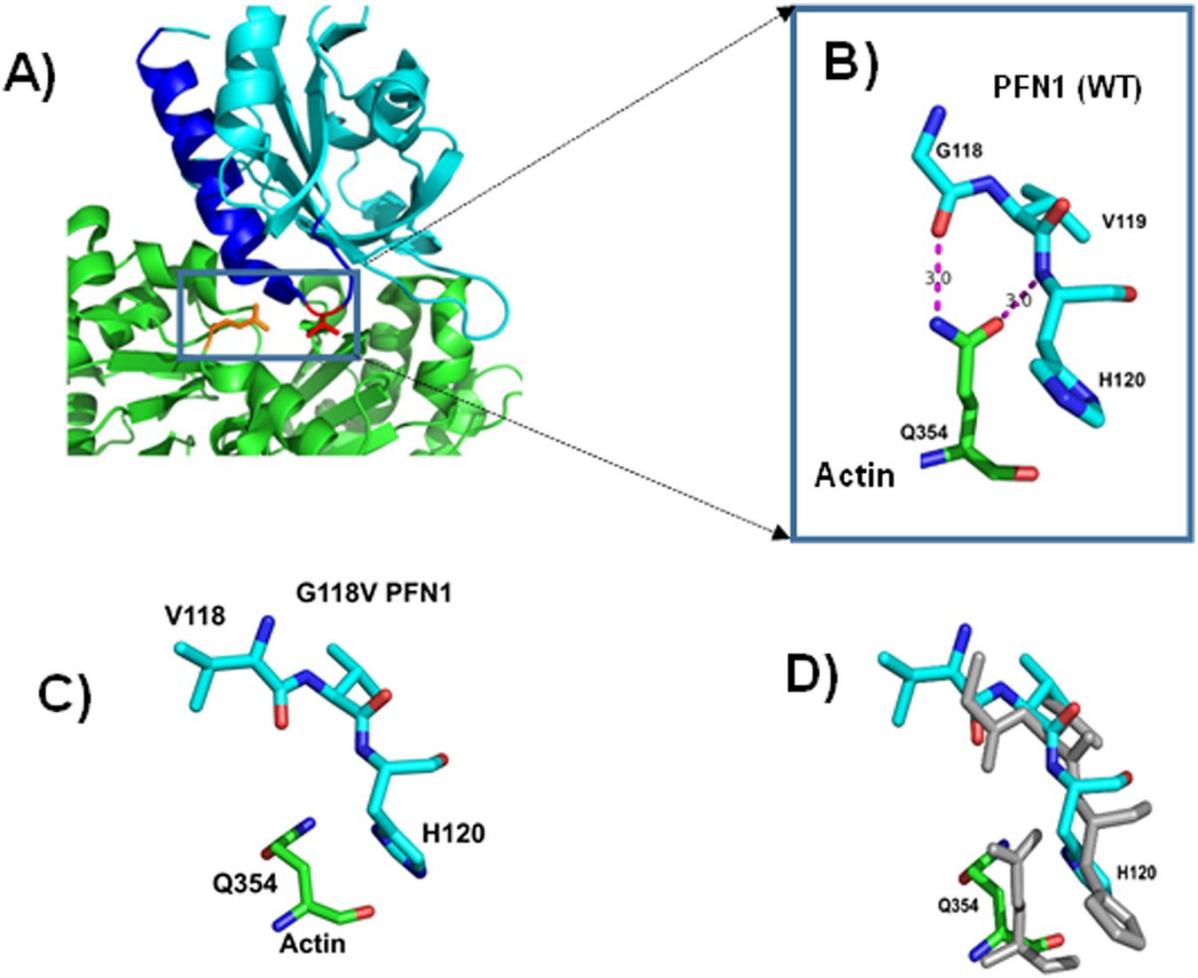
Effects of the PFN1 G118V mutation on actin binding. (**A**) Overall view of the interaction between PFN1 (cyan) and actin (green). The fifth helix and the preceding loop are colored blue; V118 is red, and Q354 of actin is yellow. **(B)** Hydrogen-bonding between actin Q354 and the G118 to H120 main-chain region of PFN1. The side chain of Q354 interacts with PFN1, forming two hydrogen bonds with the PFN1 backbone peptide chain in the loop region shown in (**A**). The nitrogen atom NE2 forms a hydrogen bond with the carbonyl oxygen of V118. The side-chain oxygen OE1 forms a hydrogen bond with the amide nitrogen of His120. The N to O distances are 3.0 Å in both cases. In addition, the H120 side chain stacks favorably on the actin surface (interactions not shown). **(C, D)** Alterations in conformation and interactions due to G118V mutation. Panel C portrays the same residues shown in (**B**) but for the PFN1^G118V^ model. The side chain of Q354 changes its orientation, eliminating the two hydrogen bonds seen in the crystal structure. Additionally, H120 also reorients disrupting interactions with action. (**D**) Superimposed wild type crystal structure (grey) with the G118V model (cyan) showing the conformational changes in the region.

The presence of a glycine residue increases the flexibility of the chain and therefore replacing Gly118 with valine would reduce the flexibility of the loop. A comparison of the Ramachandran plot for glycine^26^ with that of for amino acids with side chains shows that the presence of side chains severely restricted conformational flexibility. Therefore Gly to Val change could induce conformational changes in the peptide chain and these changes might hinder the binding of PFN1 to actin. Interestingly, the backbone peptide directly interacts via two hydrogen bonds to the side chain of Gln354 in actin (Fig. 2). The side-chain nitrogen atom NE2 formed a hydrogen bond with the carbonyl oxygen of Gly118, and the side-chain oxygen atom OE1 formed a hydrogen bond with the amide nitrogen (N) of His120. The side chain of His120 of PNF1 also plays a role in binding. It sits in a pocket surrounded by the side chains of actin amino acids Tyr169, Tyr133, Phe375, and Met355. Even minor changes in the peptide conformation could weaken or eliminate these packing interactions and hydrogen bonds. To explore the consequences of this mutation on binding, we carried out a 50-ns molecular dynamic simulation of the PFN1^G118V^: actin complex (see Methods for details). The resulting coordinates showed that the Gln354 side chain of actin moved away from the His120 side chain of PFN1, eliminating the hydrogen bonding and packing interactions (Fig. 2).

### Cumulative changes in hydrophobicity

In addition to the conformational changes described above, we hypothesized that the G118V mutation increases the local hydrophobicity of the polypeptide, the structural consequences of which would be amplified by the presence of another valine residue at position 119. We reasoned that two adjacent hydrophobic residues exposed to the solvent should be thermodynamically unfavorable and thus increase the propensity for PFN1 to aggregate. Any structural rearrangement that sequesters the valine side chains in the hydrophobic interior of PFN1 would require significant changes to the backbone conformation that might themselves preclude interactions with actin or cause misfolding and aggregation of PFN1.

### Threonine 109 to methionine mutation

**Figure 3:**
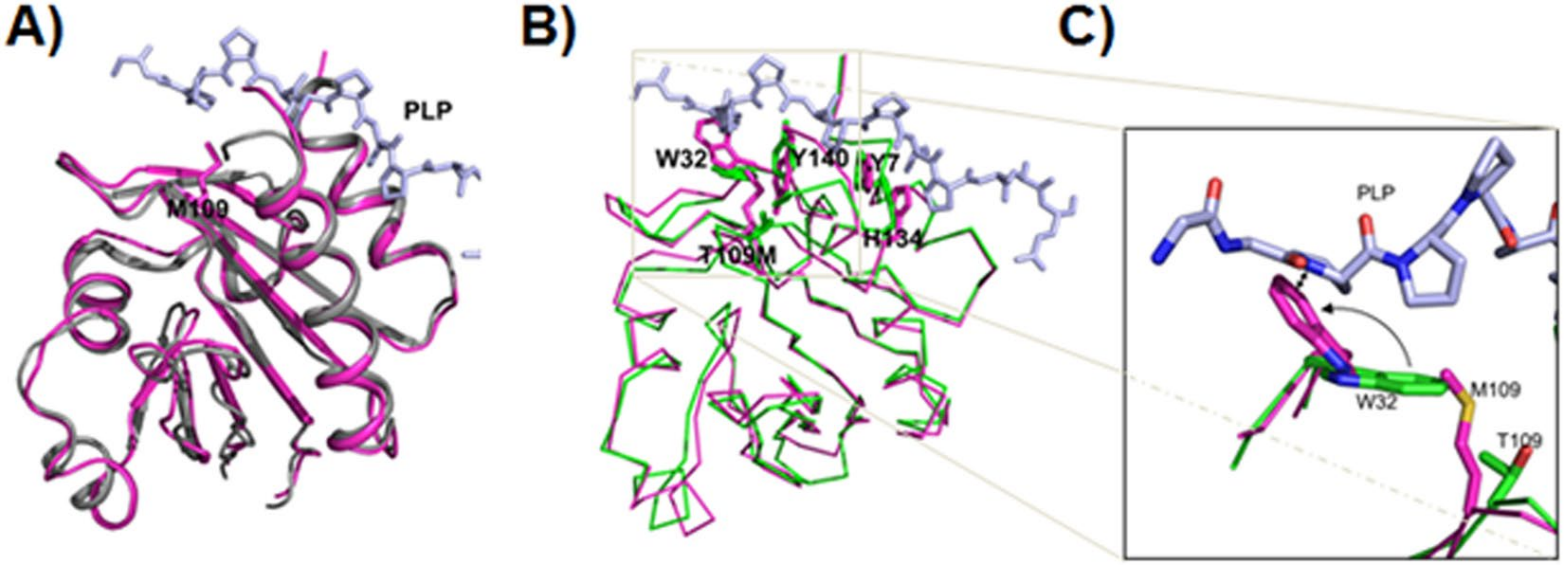
Structural consequences of the PFN1 T109M mutation. (**A**) Superimposed crystal structure of wild type PFN1 and a 200-ns simulation model of PFN1 T109M. Wild type PFN1, grey; T109M mutant, magenta; and PLP stick model, light blue. The overall structures are very similar. (**B**) Superposition of the simulated models of wild type (green) and mutant PFN1 (magenta) showing the consequences of T109M mutation on the PLP-binding site. PLP stick model, light blue. The side chains of mutated residue T109 and four other residues exhibiting large displacements (Y7, W32, H134, and Y140) at the PLP-binding site are displayed and labeled. (**C**) A maginified view of the changes around the site of mutation. The larger Met109 side-chain forces Trp32 side-chain to swing away to a new location (arched arrow) where it is too close (indicated by the double-headed arrow) to the original PLP binding location.

PFN1 also contains a binding site for PLP (Figs 1 and 3). We inferred from the co-crystalized structure that the T109M mutation could reduce the affinity of PFN1 for PLP. To assess the impact of the T109M mutation on PFN1 binding to PLP, we performed molecular-dynamic simulations. In the simulations, Met109 of PFN1 did not directly contact PLP; however, due to its close proximity to the PLP-binding site, Met109 impacted other residues that do interact with the ligand and caused structural perturbations at the binding site (Fig. 3).

To further analyze the implications of the T109M mutation, we used GROMACS simulation software^27^ to perform a 50-ns molecular-dynamics simulation with the PFN1^T109M^:PLP complex, as well as a similar simulation with the PFN1^WT^:PLP complex (see Methods for details). When we superimposed the structural models of PFN1 and the T109M mutant, the overall conformation of the mutant was almost identical to the wild type (Fig. 3A). However, a closer look at the binding site revealed that the mutation displaced the side-chains that form the PLP-binding surface (Fig. 3B). It was also evident that the large side chains of Tyr7, Trp32, Tyr140, and His134 were shifted in the mutant, changing the shape and nature of the binding site. For example, Trp32 protruded into the space occupied by the PLP peptide in the wild type (Fig. 3B). There was significant movement at the C-terminal portion, and Tyr140 was oriented in the opposite direction compared with the wild type (Fig. 3B). The most critical amino acid shift is Trp32 due to Met109 larger side-chain as we have shown in the magnified inset (Fig. 3C). These models strongly imply that the PFN1 T109M mutation might significantly reduce the affinity of PFN1 for PLP or prevent their binding altogether.

**Figure 4:**
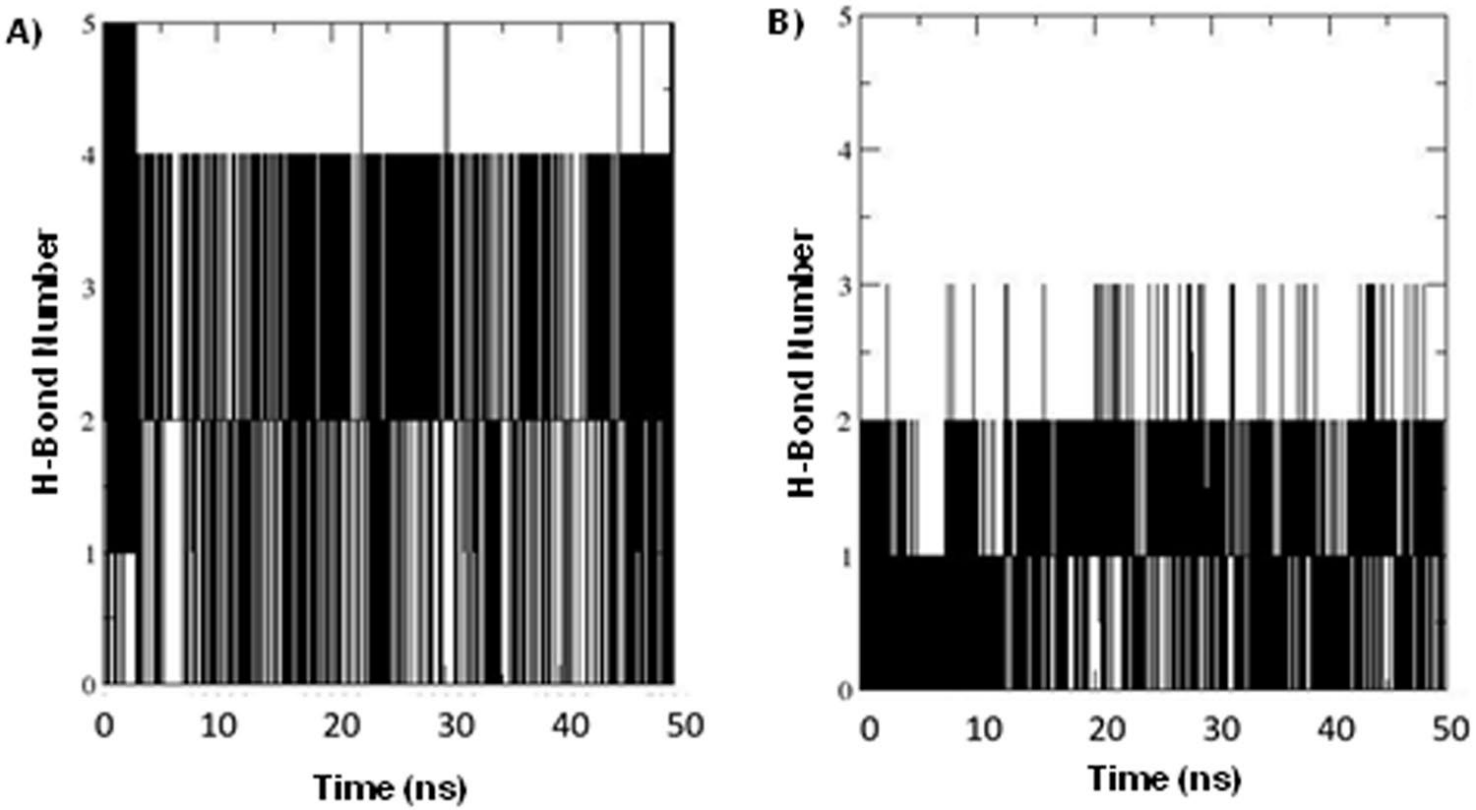
Hydrogen-bond number analysis. (**A**) The number of hydrogen bonds between PFN1^WT^ and PLP and **(B)** between PFN1^T109M^ and PLP calculated from 50-ns trajectories of the PFN1:PLP complex.

Simulations of the PFN1^WT^:PLP complex generally showed a stable association between the proteins in which hydrogen-bonding interactions between PFN1 and PLP remained constant during the simulation. However, hydrogen bonding diminished significantly between the PFN1^T109M^:PLP complex, indicating poor or unstable binding (Fig. 4). The simulation data (50 ns) indicated that the wild type maintained an average of 2.09 hydrogen bonds across the whole simulation, while the mutant maintained only 1.07 hydrogen bonds (Fig. 4). A longer simulation of 200 ns shows on average 3.30 hydrogen bonds in wild type and 3.0 in mutant PFN1 (200 ns simulation H-bond data not shown).

### Differential behavior of isolated WT and mutant PFN1

The study of tertiary and quaternary structure of PFN1 protein (wild type and mutant) in complex with actin and/or PLP requires an extensive set of molecular dynamics simulations. The simulations on PFN1 protein (wild type and mutant) in association with actin and/or PLP is considerably more complex than in the uncomplexed state. Additionally, these simulations are associated with thermal fluctuations that may not be easily distinguished from functionally relevant behaviors of a protein in different conditions (different mutations or binding states). To avoid such issues, we set out to extensively examine the behavior of isolated WT and mutant PFN1. We performed three sets of independent molecular dynamics simulations for each system including PFN1^WT^, PFN1^T109M^, and PFN1^G118V^ (three 200-ns simulations for each system).

**Figure 5:**
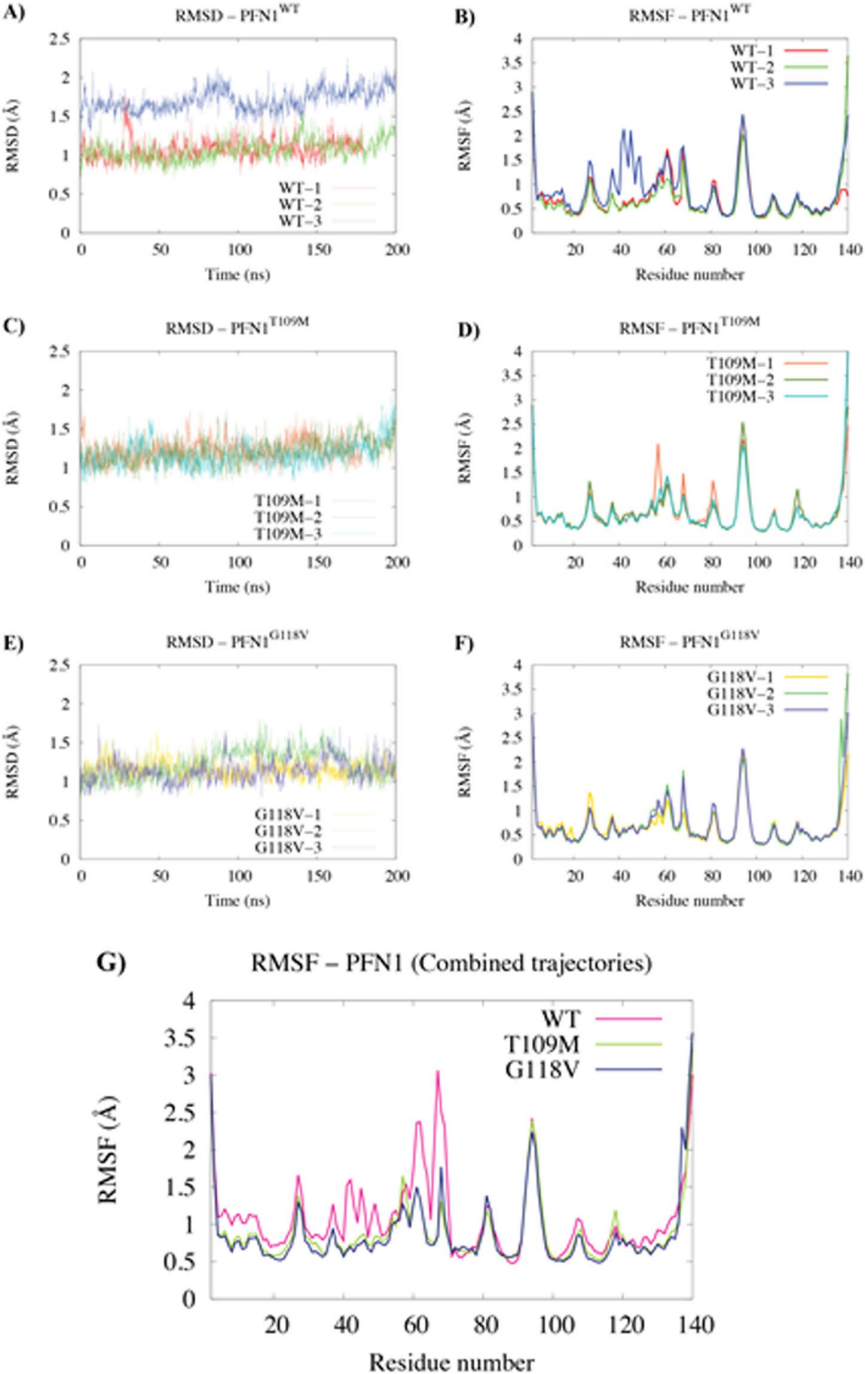
Protein fluctuations during 200 nanosecond simulations of isolated PFN1^WT^, PFN1^G118V^, and PFN1^T109M^. (**A**,**B**) Backbone root-mean-square deviation (RMSD) and residue-based C_α_ root-mean-square fluctuation (RMSF) for PFN1^WT^, **(C,D)** PFN1^T109M^, and **(E,F**) PFN1^G118V^. In each case, 3 independent 200-ns trajectories were performed and analyzed as shown in each panel. **(G)** The three trajectories of each system were then combined to generate the residue-based C_α_ RMSF shown for PFN1^WT^ (magenta), PFN1^T109M^ (green), and PFN1^G118V^ (blue).

Root-mean-square deviation (RMSD) and root-mean-square fluctuation (RMSF) analyses were performed to systematically compare the overall and residue-based fluctuations of the protein in different conditions (Fig. 5; see Methods for analysis details). In two of the three PFN1^WT^ simulations (WT-1, WT-2), the PFN1 variants behaved similarly. WT-3 simulations indicate some additional flexibility for the 35–50 region (Fig. 5G); however we cannot correlate this with any functional characteristics of the molecule without further future analysis.

### The hairpin switch mechanism of WT PFN1

**Figure 6:**
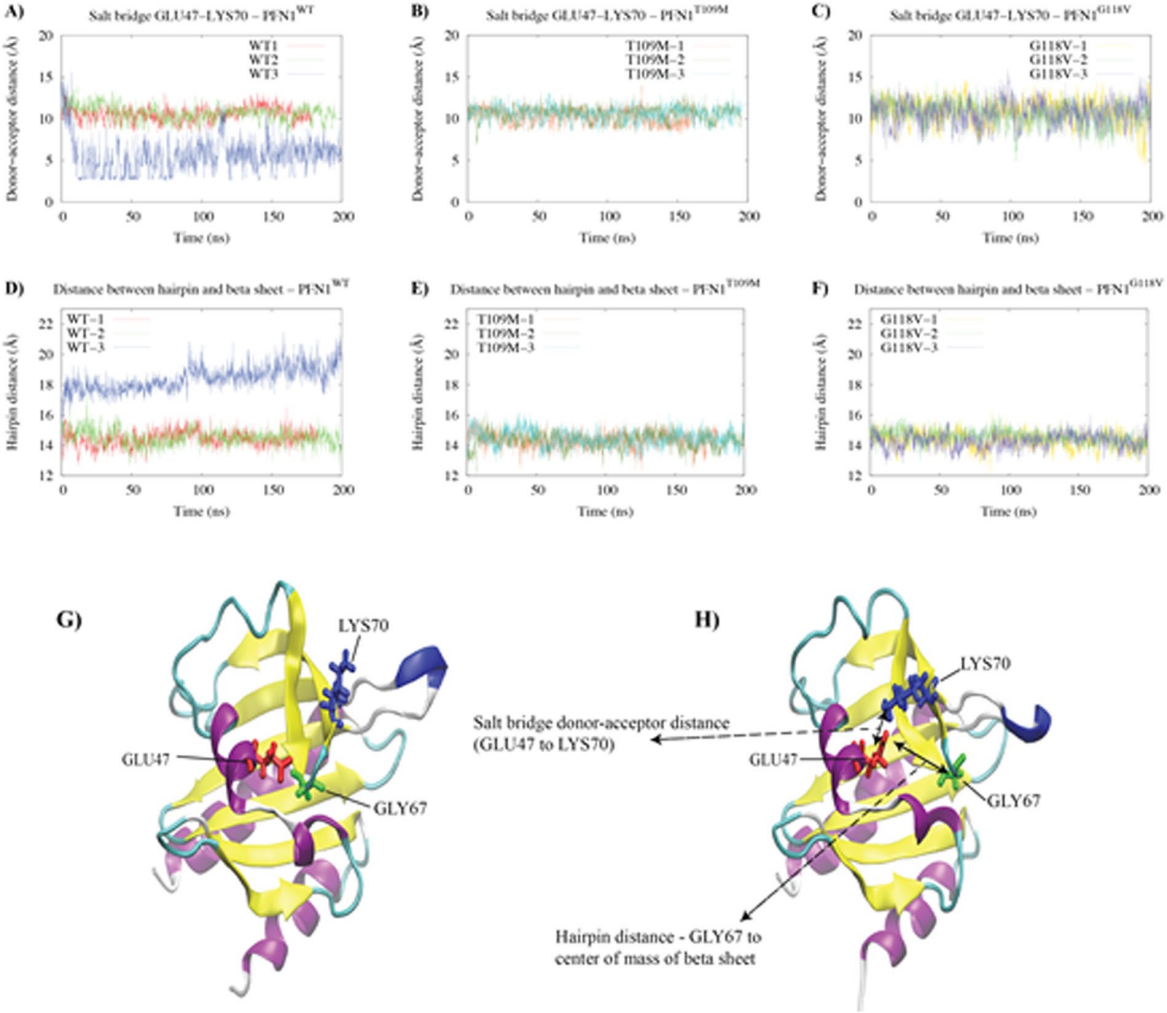
The hairpin switch mechanism of isolated PFN1^WT^. (**A**–**C**) The GLU47-LYS70 salt bridge donor–acceptor distances for isolated (**A**) PFN1^WT^, (**B**) PFN1^T109M^, and (C) PFN1^G118V^ during the 200-ns molecular dynamics simulations. **(D**–**F**) The distances between the C_α_ atom of G67 (tip of a hairpin) and the mass center of β-sheet for (**A**), PFN1^T109M^ (**B**), and PFN1^G118V^ (**C**). (**G,H**) Illustration of the GLU47-LYS70 salt bridge and twisting away of the G67 hairpin from the β-sheet (the hairpin switch mechanism) in the third Isolated PFN1^WT^ simulations by showing the initial (**G**) and final (**H**) conformations of PFN1 in cartoon representation.

A careful analysis of isolated wild type and mutant PFN1 trajectories revealed two important conformational changes, with potentially significant consequences, observed in only one of the wild type simulations (Fig. 6). An important observation was the formation of a relatively stable salt bridge between Glu47 and Lys70 (Fig. 6A–C). The salt bridge does not exist in the crystal structure of bound PFN1 and did not appear in any of the isolated mutant simulations. The second observation was that a particular hairpin twists away from the protein (Fig. 6D–F). Figure 6G,H illustrates the simultaneous formation of the Glu47-Lys70 salt bridge and twisting away of the G67 hairpin from the PFN1 β-sheet. The hairpin twist occurs just before the formation of the salt bridge (Fig. 6A,D), indicating that the salt bridge might be formed by the displacement of the Gly67 hairpin, which also moves Lys70 with it. We suggest that the conformation of PFN1 observed in the third wild type simulation might more accurately describe the conformation of isolated PFN1. In other words, wild type PFN1 might have a switch mechanism to adopt different conformations when the protein is free or bound.

### Rigidity of the actin/PLP binding site in mutant PFN1

Both of the mutant PFN1 proteins simulated here appear to be associated with more rigid binding sites and cannot adopt the conformation of isolated wild type PFN1. This will eventually result in other conformational changes that are beyond the timescales of the simulations performed but that would lead to the misfolding and aggregation of PFN1. We thus hypothesize that the observed rigidity of the binding sites in the G118V and T109M mutants diminishes the binding of actin and PLP and eventually causes more dramatic conformational changes that increase the propensity of PFN1 for aggregation.

**Figure 7:**
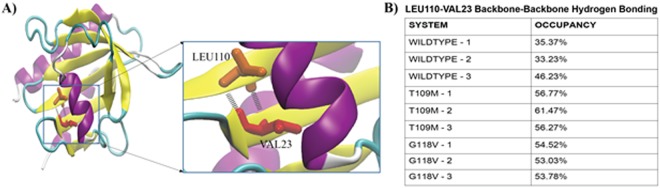
Backbone hydrogen-bonding analysis of PFN1. (**A**) Illustration of the backbone hydrogen bond between Leu110 and Val23 shown using cartoon representation of PFN1. (**B**) The Leu110-Val23 backbone hydrogen bond occupancy estimated from individual 200-ns molecular dynamics trajectories of isolated PFN1.

We specifically analyzed the hydrogen-bonding pattern in the wild type and mutant simulations. Certain backbone hydrogen bonds seemed to be consistently more stable in the mutant PFN1 than in wild type. An example is the hydrogen bond between the backbones of Leu110 and Val23 (Fig. 7). The occupancy of this hydrogen bond was always greater than 50% in the mutant PFN1 simulations (for both G118V and T109M mutants), while it was always smaller than 50% in the wild type PFN1 simulations. This observation is consistent with the rigidity hypothesis for mutant PFN1.

## Discussion

A number of mutations in *PFN1* have been functionally linked to neurodegeneration in fALS^1,2,5^, but the mechanism of PFN1 neurotoxicity remains undetermined. The locations of fALS-associated mutations in PFN1, considered in conjunction with its known interactions, suggest that PFN1 neurotoxicity may involve two distinct pathways, actin-binding dynamics and the PLP-binding signaling pathway, that result in the degeneration of motor neurons^1,2,5,19,20,28–31^.

The data and analyses described here provide novel insights into two putative mechanisms: the disruption of interactions between PFN1 and actin- or PLP-binding motifs. We have presented evidence that the G118V mutation changes local hydrophobicity and reduces the flexibility of the actin-interacting loop, which could explain the observed reduction in actin polymerization and the perturbed equilibrium and reduced ratios of F-actin to G-actin^1,2,5^. The G118V mutation is adjacent to the actin-binding interface, and the additional conformational rigidity arising from the Gly to Val mutation causes conformational changes that prevent favorable interactions with actin. In addition, it is possible that the fifth helix of PFN1 gets elongated under the influence of this mutation and the consequences of this are discussed below.

It has been widely observed that alpha-helical regions are terminated by prolines. Glycine is not generally viewed as a helix-terminating residue, but it serves as a suitable amino acid to form bends and kinks in tertiary protein structures. Presuming that Gly118 helps form the bend and because it terminates the fifth alpha-helix in PFN1, then our hypothesis gains further support from the possibility that when Gly118 is changed to valine, the helix will be extended. Any such change will negatively impact binding because the polypeptide region immediately following the helix interacts strongly with actin. Circular dichroism (CD) and other physical characterizations of the G118V mutation support this notion, as described below.

In a concurrent study of PFN1 structural characteristics, an analysis of PFN1 with CD showed a spectral shift, indicating a conformational change by extension of the alpha-helix (Nekouie *et al*., 2018, manuscript under review). This observation suggests that the mutation of Gly118 is pivotal for proper folding to generate the proper geometry for interactions with actin. In addition, the changes caused by the substitution of glycine with valine may lead to the misfolding and aggregation of the protein. The T109M mutation, on the other hand, appears to distort the PLP-binding site so as to make it sterically unsuitable for PLP binding. Our simulation studies showed that the mutant is unable to maintain the hydrogen-bonding interactions with PLP. We observed that during simulation, wild type PNF1 maintains its hydrogen bonds with PLP, while the hydrogen-bonding interactions decrease sharply for the T109M mutant.

Our analyses indicate that the G118V and T109M mutations increase the rigidity of surrounding regions, impairing actin- and PLP-binding, respectively. In addition, the simulation of WT PFN1 produced an unexpected finding in that it exhibits conformational isomerism. Our analysis of isolated WT PFN1 trajectories during simulations showed that a hairpin loop containing Gly67 at the tip can twist away from the PFN1 β-sheet, and a relatively stable salt bridge between the Glu47 and Lys70 residues is formed. We did not observe similar results for either of the mutants, and we postulate that this may have some functional relevance, as PNF1 is a multi-functional protein that interacts with several ligands. Therefore, the conformational isomerism and additional flexibility observed in wild type PFN1 may be necessary for its diverse roles.

In conclusion, our *in silico* study revealed that mutant PFN1 may gain propensity for aggregation due to weakening or loss of hydrogen bonds and packing interactions, significant change in the backbone conformation, poor and unstable binding at PLP region and salt bridge formation leading to rigidity of actin/PLP binding site in mutant PFN1.

## Methods

### Mutant structure generation

A crystallographic structure of native PFN1 was retrieved from PDB databank (2PAV). This structure was used to create G118V and T109M mutations using the mutagenesis plugin within Pymol (www.pymol.org). Mutant structures and the native form of PFN1 were energy-minimized in a solvent box with counter-ions and 0.14 M NaCl for 5000 steps using the “steepest decent” method in GROMACS molecular dynamics simulation software; we used the Assisted Model Building and the Energy Refinement (AMBER99SB-ILDN) force field that refers to both sets of molecular mechanical force fields to simulate biomolecules and uses a package of molecular simulation programs. For more details, refer to http://www.gromacs.org.

### Atomistic molecular dynamics simulations of isolated PFN1

To understand the impact of single amino acid (missense) mutations on PFN1 structure, molecular dynamics simulations were performed in explicit solvent for PFN1^WT^, PFN1^T109M^, and PFN1^G118V^. Energy-minimized protein structures were immersed in a cubic box containing SPC216 solvent-containing counter ions. Further, 0.15 M NaCl (to achieve an ionic strength similar to blood) was added to crudely mimic physiological conditions. The AMBER99SB-ILDN force field was used for all simulations. The system was further energy-minimized for 5000 steps using the steepest descent method. The system was then equilibrated at constant temperature (300 K), in the NVT ensemble (i.e., constant number of particles, volume, and temperature) for 300 ps. Next, the system was equilibrated at constant pressure (1 bar) in the NPT ensemble (i.e., constant particle number, pressure, and temperature). The final simulation (production) was carried out for each system for 200 ns and was repeated 3 times (three independent simulations for each system). The leap-frog integrator was used for the time evolution of trajectories.

### Analysis of isolated PFN1 MD trajectories

The resulting trajectories were analyzed with trajectory analysis modules from the GROMACS simulation package and from Visual Molecular Dynamics (VMD) software. Multiple VMD analyses were performed on the trajectories from nine 200-ns PFN1 simulations, including RMSD (Root-Mean-Square Deviation - backbone) and RMSF (Root-Mean-Square Fluctuation – C_α_). Hydrogen-bonding was analyzed with the Hydrogen Bonds plugin in VMD. Detailed information was generated for all hydrogen bonds in each system using a donor-acceptor cutoff distance of 3 A° and an angle cutoff of 20°. Salt bridges were analyzed with the Salt Bridges plugin in VMD. For each system, a donor-acceptor cutoff distance of 4 A° was used to generate data for individual salt bridges (distances over time). Each salt bridge was plotted to assess the strength of the interaction.

### Disclousure Statement

Dr. Kiaei and UAMS have a financial interest in the technology discussed in this publication. These financial interests have been reviewed and approved in accordance with the UAMS conflict of interest policies.
